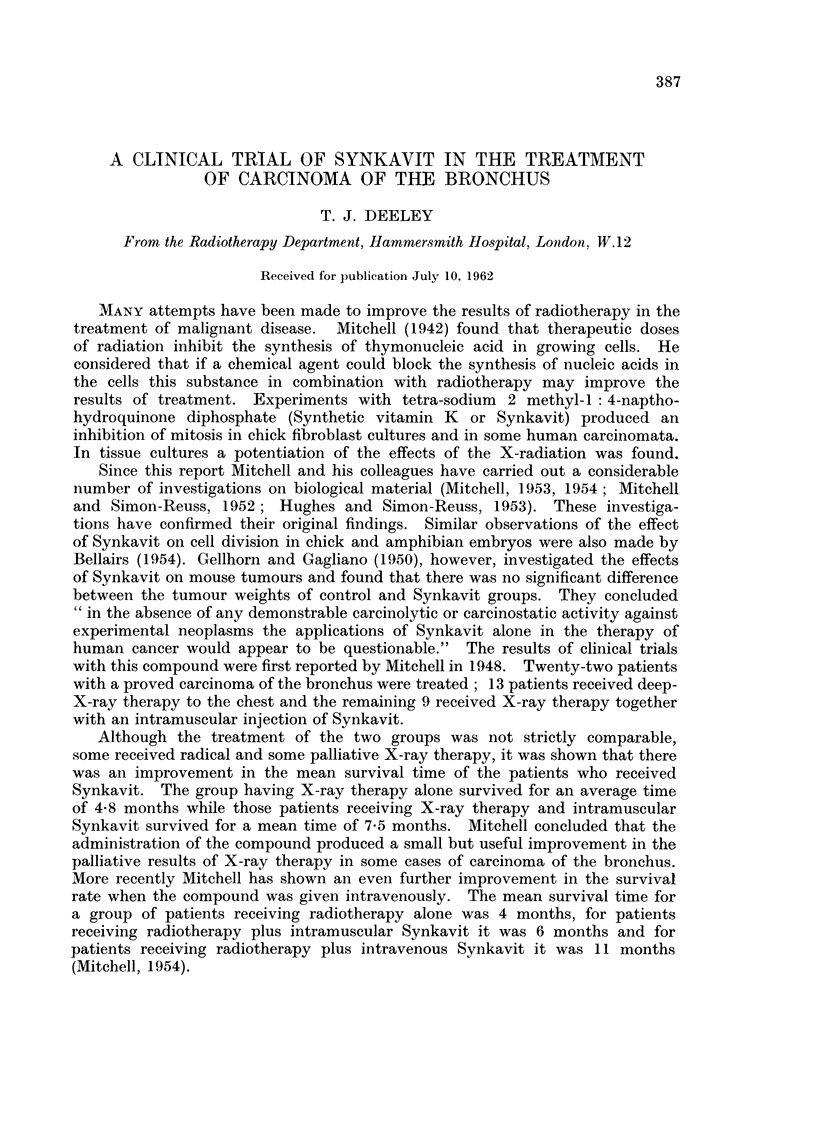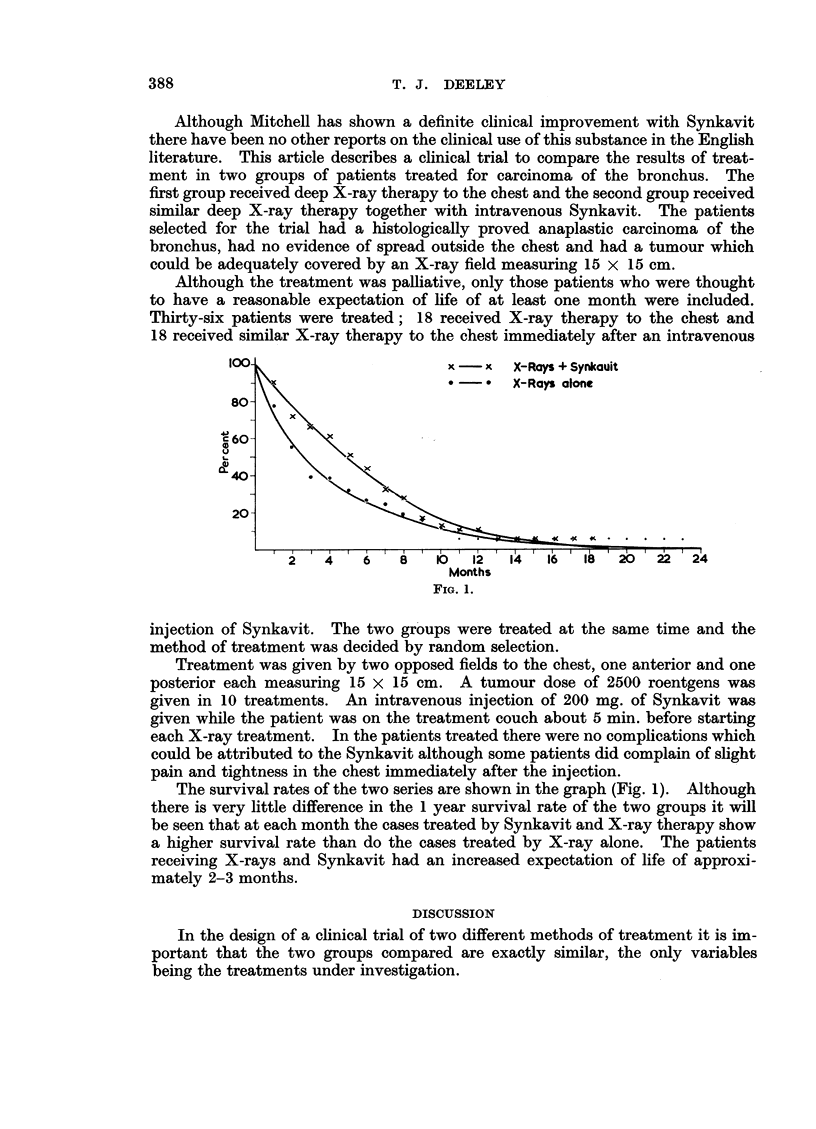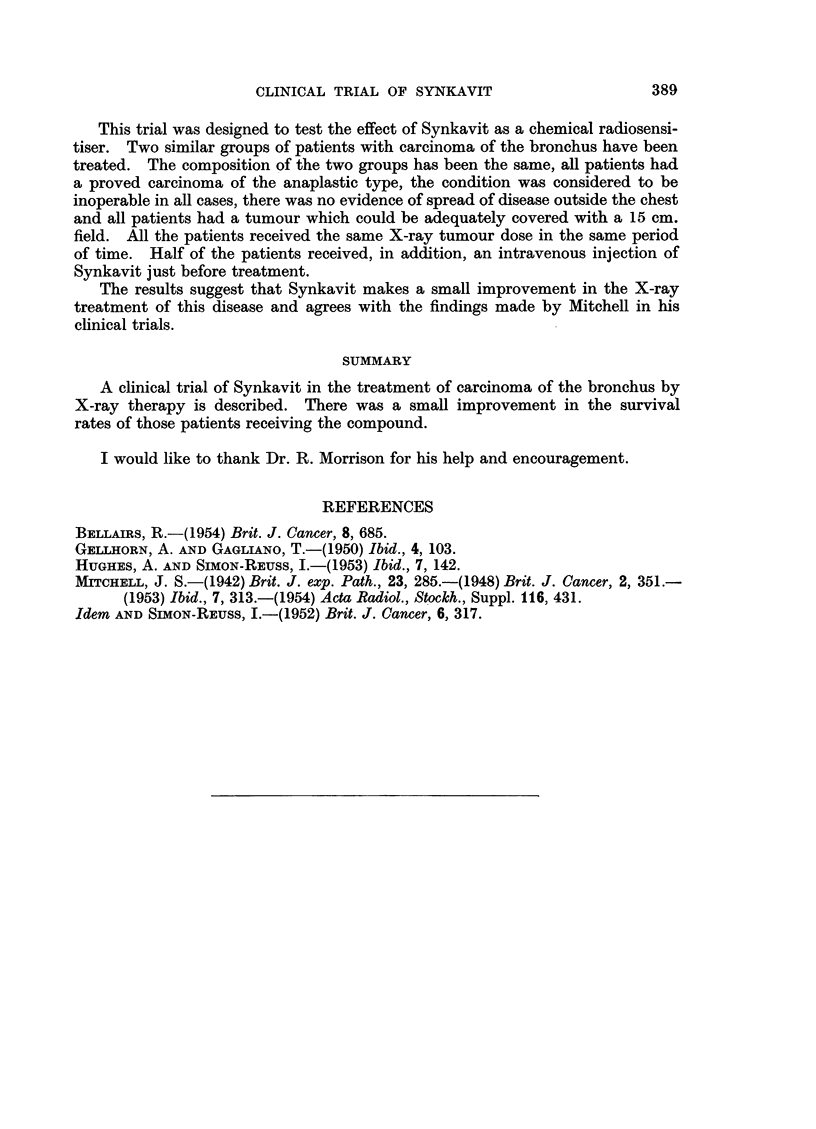# A Clinical Trial of Synkavit in the Treatment of Carcinoma of the Bronchus

**DOI:** 10.1038/bjc.1962.44

**Published:** 1962-09

**Authors:** T. J. Deeley


### A CLINICAL TRIAL OF SYNKAVIT IN THE TREATMENT OF CARCINOMA OF THE BRONCHUS

T. J. DEELEY

*From the Radiotherapy Department, Hammersmith Hospital, London, W.12*

Received for publication July 10, 1962

MANY attempts have been made to improve the results of radiotherapy in the treatment of malignant disease. Mitchell (1942) found that therapeutic doses of radiation inhibit the synthesis of thymonucleic acid in growing cells. He considered that if a chemical agent could block the synthesis of nucleic acids in the cells this substance in combination with radiotherapy may improve the results of treatment. Experiments with tetra-sodium 2 methyl-1 : 4-naptho-hydroquinone diphosphate (Synthetic vitamin K or Synkavit) produced an inhibition of mitosis in chick fibroblast cultures and in some human carcinomata. In tissue cultures a potentiation of the effects of the X-radiation was found.

Since this report Mitchell and his colleagues have carried out a considerable number of investigations on biological material (Mitchell, 1953, 1954 ; Mitchell and Simon-Reuss, 1952 ; Hughes and Simon-Reuss, 1953). These investigations have confirmed their original findings. Similar observations of the effect of Synkavit on cell division in chick and amphibian embryos were also made by Bellairs (1954). Gellhorn and Gagliano (1950), however, investigated the effects of Synkavit on mouse tumours and found that there was no significant difference between the tumour weights of control and Synkavit groups. They concluded " in the absence of any demonstrable carcinolytic or carcinostatic activity against experimental neoplasms the applications of Synkavit alone in the therapy of human cancer would appear to be questionable." The results of clinical trials with this compound were first reported by Mitchell in 1948. Twenty-two patients with a proved carcinoma of the bronchus were treated ; 13 patients received deep-X-ray therapy to the chest and the remaining 9 received X-ray therapy together with an intramuscular injection of Synkavit.

Although the treatment of the two groups was not strictly comparable, some received radical and some palliative X-ray therapy, it was shown that there was an improvement in the mean survival time of the patients who received Synkavit. The group having X-ray therapy alone survived for an average time of 4·8 months while those patients receiving X-ray therapy and intramuscular Synkavit survived for a mean time of 7·5 months. Mitchell concluded that the administration of the compound produced a small but useful improvement in the palliative results of X-ray therapy in some cases of carcinoma of the bronchus. More recently Mitchell has shown an even further improvement in the survival rate when the compound was given intravenously. The mean survival time for a group of patients receiving radiotherapy alone was 4 months, for patients receiving radiotherapy plus intramuscular Synkavit it was 6 months and for patients receiving radiotherapy plus intravenous Synkavit it was 11 months (Mitchell, 1954).

Although Mitchell has shown a definite clinical improvement with Synkavit there have been no other reports on the clinical use of this substance in the English literature. This article describes a clinical trial to compare the results of treatment in two groups of patients treated for carcinoma of the bronchus. The first group received deep X-ray therapy to the chest and the second group received similar deep X-ray therapy together with intravenous Synkavit. The patients selected for the trial had a histologically proved anaplastic carcinoma of the bronchus, had no evidence of spread outside the chest and had a tumour which could be adequately covered by an X-ray field measuring 15 × 15 cm.

Although the treatment was palliative, only those patients who were thought to have a reasonable expectation of life of at least one month were included. Thirty-six patients were treated; 18 received X-ray therapy to the chest and 18 received similar X-ray therapy to the chest immediately after an intravenous

Fig. 1.

injection of Synkavit. The two groups were treated at the same time and the method of treatment was decided by random selection.

Treatment was given by two opposed fields to the chest, one anterior and one posterior each measuring 15 × 15 cm. A tumour dose of 2500 roentgens was given in 10 treatments. An intravenous injection of 200 mg. of Synkavit was given while the patient was on the treatment couch about 5 min. before starting each X-ray treatment. In the patients treated there were no complications which could be attributed to the Synkavit although some patients did complain of slight pain and tightness in the chest immediately after the injection.

The survival rates of the two series are shown in the graph (Fig. 1). Although there is very little difference in the 1 year survival rate of the two groups it will be seen that at each month the cases treated by Synkavit and X-ray therapy show a higher survival rate than do the cases treated by X-ray alone. The patients receiving X-rays and Synkavit had an increased expectation of life of approximately 2–3 months.

### DISCUSSION

In the design of a clinical trial of two different methods of treatment it is important that the two groups compared are exactly similar, the only variables being the treatments under investigation.

This trial was designed to test the effect of Synkavit as a chemical radiosensitiser. Two similar groups of patients with carcinoma of the bronchus have been treated. The composition of the two groups has been the same, all patients had a proved carcinoma of the anaplastic type, the condition was considered to be inoperable in all cases, there was no evidence of spread of disease outside the chest and all patients had a tumour which could be adequately covered with a 15 cm. field. All the patients received the same X-ray tumour dose in the same period of time. Half of the patients received, in addition, an intravenous injection of Synkavit just before treatment.

The results suggest that Synkavit makes a small improvement in the X-ray treatment of this disease and agrees with the findings made by Mitchell in his clinical trials.

### SUMMARY

A clinical trial of Synkavit in the treatment of carcinoma of the bronchus by X-ray therapy is described. There was a small improvement in the survival rates of those patients receiving the compound.

I would like to thank Dr. R. Morrison for his help and encouragement.

### REFERENCES

Bellairs, R.—(1954) *Brit. J. Cancer*, **8**, 685.
Gellhorn, A. and Gagliano, T.—(1950) *Ibid.*, **4**, 103.
Hughes, A. and Simon-Reuss, I.—(1953) *Ibid.*, **7**, 142.
Mitchell, J. S.—(1942) *Brit. J. exp. Path.*, **23**, 285.—(1948) *Brit. J. Cancer*, **2**, 351.—(1953) *Ibid.*, **7**, 313.—(1954) *Acta Radiol., Stockh.*, Suppl. **116**, 431.
*Idem* and Simon-Reuss, I.—(1952) *Brit. J. Cancer*, **6**, 317.